# Genetic landscape of patients with atypical absence status epilepticus: A systematic review

**DOI:** 10.1002/epi4.70275

**Published:** 2026-05-11

**Authors:** Maria Cristina Cioclu, Giada Giovannini, Stefano Meletti

**Affiliations:** ^1^ Department of Biomedical, Metabolic, and Neural Sciences University of Modena and Reggio Emilia Modena Italy; ^2^ Neurophysiology Unit and Epilepsy Centre Azienda Ospedaliera‐Universitaria di Modena Modena Italy

**Keywords:** AASE, absence status epilepticus, DEE, genetic epilepsy, NCSE

## Abstract

Atypical absence status epilepticus (AASE) is a rare subtype of nonconvulsive status epilepticus (NCSE), characterized by clouding of consciousness and continuous or fluctuating epileptiform activity, generally at a frequency below 3 Hz. Only sparse literature exists on the genetic conditions associated with it. We performed a systematic review to identify the genetic diagnoses in which AASE has been described, and examined the available clinical, EEG, treatment and outcome details. The protocol was registered in PROSPERO and reporting followed PRISMA guidelines. We searched public databases for combinations of the terms: “atypical absence status epilepticus,” “status epilepticus,” “non convulsive status epilepticus,” and “gene,” “genetics,” “epileptic encephalopathy,” “developmental and epileptic encephalopathy,” “DEE.” Only original articles in English were included. We identified 34 publications reporting 97 patients with AASE and an underlying genetic condition. Most patients had a chromosomal abnormality (88%), in particular ring chromosome 20 (53% of the total patients) and Angelman syndrome caused by a 15q11–q13 deletion (31%). Seven epilepsy genes (*UBE3A*, *CNKSR2*, *TRPM3*, *KCNH2*, *NEXMIF*, *SYNGAP1*, *GABRB1*) were found in which a clinical and electrographic picture consistent with AASE was described. Therefore, in the context of a possible genetic diagnosis, AASE has been reported mainly in chromosomal disorders. However, this condition is likely underrecognized and underreported, particularly in monogenic epilepsies. Therefore, a deeper phenotyping and a more standardized use of classification terms would be necessary both for clinical and research purposes.

**Plain Language Summary:**

We reviewed the scientific literature to find out in which genetic conditions a rare EEG and clinical pattern, called atypical absence status epilepticus, has been described. We found that this pattern is mainly reported in patients with changes in chromosome structure, such as ring chromosome 20 and Angelman syndrome. Among single‐gene (monogenic) forms of epilepsy, it has been described in association with seven genes (*UBE3A*, *CNKSR2*, *TRPM3*, *KCNH2*, *NEXMIF*, *SYNGAP1*, *GABRB1*). When clinicians suspect a genetic cause of epilepsy and this finding is present, they should consider checking also for chromosomal changes.

**Key points**

- AASE is a rare NCSE subtype, likely underdiagnosed and underreported.
- In genetic disorders, most reported AASE cases involve chromosomal abnormalities (88%).
- Ring chromosome 20 (53%) and Angelman syndrome (35%) are the most common conditions linked to AASE.
- AASE electroclinical pattern was reported in seven genes: *UBE3A*, *CNKSR2*, *TRPM3*, *KCNH2*, *NEXMIF*, *SYNGAP1*, *GABRB1*.

## 1 INTRODUCTION

Nonconvulsive status epilepticus (NCSE) represents an electroclinical condition characterized by prolonged (more than 10 min) nonconvulsive seizure activity, either continuous or recurrent, without a return to baseline conditions between seizures.^1^ The clinical presentation can be heterogeneous and can include sensory, cognitive, behavioral, and autonomic features. Subtle motor phenomena such as minor muscular twitches may be present as well. Since the diverse and nonspecific clinical aspects, the diagnosis of this condition relies on a high degree of suspicion and on the EEG confirmation of continuous ictal activity.^1^, ^2^, ^3^

The in‐depth characterization of the clinical and electrographic features of seizures and status epilepticus, hence their correct classification, represents one of the first steps in setting up the appropriate diagnostic work‐up and treatment plan for each patient.^3^, ^4^, ^5^

Atypical absence status epilepticus (AASE) is a subtype of NCSE with different degrees of impaired consciousness (without coma), which has mainly been described in children with severe epileptic encephalopathies, usually in association with developmental delay and intellectual disability, such as Lennox‐Gastaut syndrome, myoclonic–static epilepsy, Angelman syndrome, and ring chromosome 20.^3^, ^6^, ^7^, ^8^

The EEG shows continuous or waxing and waning epileptic abnormalities, mainly slow spike‐ polyspike‐and‐wave activity, and generally at a frequency of less than 3 Hz.^7^ The clinical manifestations of this type of status are often difficult to recognize, since, in many cases, they present only as a worsening of the underlying neurological condition (e.g., a reduction in motor activity, an increase in drowsiness or an aggravation of behavioral issues). Moreover, the onset and termination of status may be gradual and therefore less easily perceived. For these reasons, the diagnosis of AASE is often not straightforward and prolonged video‐EEG monitoring, showing a significant variation from baseline activity, is generally required for accurate identification and management.^6^, ^9^, ^10^

The purpose of this study is to review AASE associated with genetic conditions, summarize patients' general clinical features, and review reported treatments and outcomes when available. Moreover, we aimed to establish an initial framework to evaluate whether the occurrence of this SE subtype could ultimately inform decisions on genetic testing and aid interpretation of genetic findings.

## 2 METHODS

The results of this systematic review were reported according to the recommendations of the Preferred Reporting Items for Systematic Reviews and Meta‐Analyses (PRISMA) statement and the Synthesis Without Meta‐analysis in systematic reviews (SWiM) extension.^11^, ^12^ The protocol was registered in PROSPERO (CRD420261331248).

The relevant studies were identified through MEDLINE (accessed by PubMed) and EMBASE up to February 15, 2026.

The search terms were combinations of the following: “atypical absence status epilepticus,” “status epilepticus,” “non convulsive status epilepticus,” “non‐ convulsive status epilepticus,” “NCSE,” “non‐convulsive” and “gene,” “genetics,” “epileptic encephalopathy,” “developmental and epileptic encephalopathy,” “DEE,” “genetic epilepsy” in various combinations. The complete search strategy is outlined in Data S1.

The following types of studies were considered for inclusion: cohorts, case–control, cross‐sectional, clinical series, and case reports. Self‐reported surveys, reviews/meta‐analyses, editorials, letters to the editor, commentaries, abstracts, and expert opinions were excluded. Also, we have excluded the articles on animal models or in vitro studies. Only articles written in the English language were considered. Participants of any age, sex, and ethnicity were eligible.

### 2.1 Study definitions

#### 2.1.1 AASE

A subtype of NCSE characterized by different degree of impaired consciousness (without coma), associated with slow generalized spike‐ or polyspike‐and‐wave activity, typically at a frequency of less than 3 Hz.^3^, ^7^, ^8^ According to the ILAE 2015 classification of status epilepticus, this condition is classified as NCSE without coma (generalized), AASE.^3^

#### 2.1.2 Genetic etiology and EEG patterns

We included patients with a genetic etiology confirmed by molecular or cytogenetic testing. Only variants reported as pathogenic or likely pathogenic were included.

We found a lack of concordance across studies in the classification of similar EEG patterns, some defining them AASE, while others leaning more toward NCSE or “complex partial status epilepticus” (CPSE). We therefore decided to include, for the purpose of this study, not only the articles which specifically mentioned AASE but also the ones describing patients with NCSE without coma, with generalized EEG features, without a clear‐cut focus identified clinically or on the EEG (predominance of epileptic discharges in bilateral frontal or fronto‐temporal regions was allowed).

Reported patients were excluded from subsequent analyses when clinical and EEG information was insufficient to characterize the status epilepticus subtype and when a genetic diagnosis was not provided.

We also excluded cases where either the EEG showed regular, continuous, generalized spike–wave at 3–4 Hz, in the context of idiopathic generalized epilepsy, consistent with a diagnosis of typical absence status epilepticus, or the authors themselves classified the status as typical absence status epilepticus. Cases with a prominent myoclonic component, configuring a myoclonic status epilepticus were also not considered. We did however include articles describing patients with absence status with eyelid myoclonia or subtle myoclonia.

Two authors (MCC, GG) independently assessed studies for inclusion using Rayyan,^13^ and any disagreement was resolved by discussion with a third senior author (SM). Data were extracted using a predefined standardized form by two authors (MCC, GG), and disagreements were resolved by discussion with a third senior author (SM). We did not contact study investigators for additional information. No automation tools were used.

The following information was extracted: first author and year of publication, number and demographics of participants, etiologies identified in individual patients, clinical features, reported treatments and outcomes when available.

Data were extracted at the patient level whenever possible. Ages reported in months/years were converted to a common unit for descriptive statistics. EEG description data were extracted as reported. Missing information was recorded as not available (NA).

Results were summarized using descriptive tables. Figures were used to display the PRISMA diagram, the distribution of genetic etiologies, and to provide an illustrative testing workflow.

## 3 RESULTS

**FIGURE 1:**
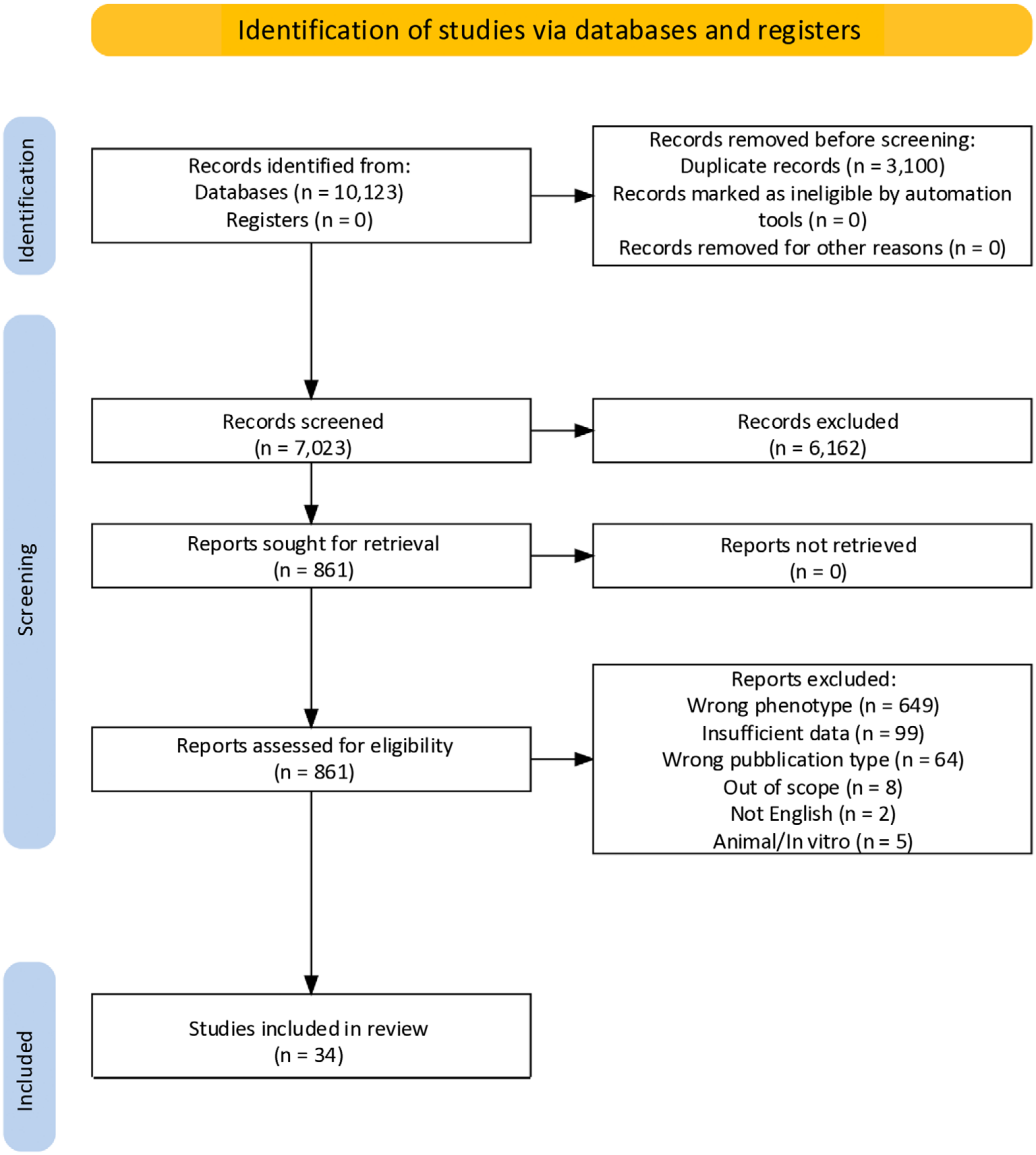
PRISMA flow diagram of the review process.

We identified 34 publications^14^, ^15^, ^16^, ^17^, ^18^, ^19^, ^20^, ^21^, ^22^, ^23^, ^24^, ^25^, ^26^, ^27^, ^28^, ^29^, ^30^, ^31^, ^32^, ^33^, ^34^, ^35^, ^36^, ^37^, ^38^, ^39^, ^40^, ^41^, ^42^, ^43^, ^44^, ^45^, ^46^, ^47^ reporting genetic variants associated with AASE (Figure 1 and Table 1 and Data S2).

**TABLE 1:** Summary of reviewed studies.

Nr	Gene/CNV	Syndr.	Publication (author, year)	Nr pts	Def. in paper	Age at first AASE	Age SE observed/last SE described	EEG status	Clinical semiology AASE	Trigger of AASE	Treatment of AASE	Outcome of SE
1	del15q12	Angelman Syndrome	Matsumoto 1992	4	AASE	NA, NA, 8y, 6y2mo	NA, NA, 10 yrs, 6y8mo	Cont. Diff. 2‐3 Hz SW bursts	Subseq AA (5‐6 sec) with rhythmic E‐my	NA	DZP, TRH	2/4 episodes resp to DZP, 1/4 resp to TRH, 1/4 NA
2	del15q11‐13	Angelman Syndrome	Sugimoto, 1992	1	Minor epileptic status	38 mo	38 mo	Freq irreg. SW	Falls, unable to use hands, ic, drooping eyelids, hypersaliv.	NA	CNZ	Marked improv.
3	del15q11‐13	Angelman Syndrome	Laan, 1997	3	ASE	NA	NA	NA	NA	NA	NA	NA
4	del15q11‐13	Angelman Syndrome	Minassian, 1998	4	AASE	NA ‐ 36 mo ‐ 15 mo ‐ 12 mo	NA ‐ 36 mo ‐ 15 mo ‐ 12 mo	NA	NA	1 pt PHT and CBZ	NA	NA
5	UBE3A c.2251insAACTA	Angelman Syndrome	Espay, 2005	1	AASE	19y	29 y	Bursts (0,2‐2,5 sec) rhyth. α, F predom.	Change in behav, head ext., E‐my, up. eye dev., arrest of UL stereotyp	no	NA	Resolved after max 2 hours
6	del15q11‐13*	Angelman Syndrome	Uemura, 2005	10	AASE	NA	15 y, 6 y, 6 y, 11 y, 6 y, 7 y, 1 y, 3 y, 2 y, 1 y	NA	NA	NA	NA	NA
7	del15q11‐13*	Angelman Syndrome	Valente, 2006	8	AASE	5 mo (in 1 pt)	NA	NA	Imp. contact, head dropping or trembling	CBZ in 1 pt, fever in 2 pts	NA	NA
8	UBE3A c.2365del	Angelman syndrome	Melikishvili, 2022	2	NCSE	66 mo, 49 mo	66 mo, 49 mo	Pt. 1: cont. 1.5–2 Hz S‐polyS‐SlW; pt. 2: cont. Diff. ↑‐volt S‐polyS‐SlW	Pt. 1: bad mood, not resp, poor sleep; pt. 2: ↓ alertness, loss of eye contact, ↓ motor activity	No	IV pyridoxine, BDZ, VPA, KD	Resolved with KD
9	r(20)	Ring chromosome 20	Inoue, 1997	3	NCSE/CPSE	14 y, NA, 7 y	21 y, 15 y, 31 y	Irreg. ↑‐volt. SlW, occasional S	Clouding of consciousness	HV for 1 pt	Lidocain in one pt, rest NA	Resolved
10	r(20)	Ring Chromosome 20	Petit, 1999	3	ASE	9 y, 5 y, 4 y	NA, NA, 43 y	Pt. 1: cont. Diff. Sh theta or SW (>biF); pt 2: fluct. Rhythmic SlW; pt 3 ↑Volt SlW+occasional S	Pt 1: loss of contact and motor automatisms; pt 2: confusion, fright, perioral my; pt 3: ↓ motor and verbal spontaneity, peiroral my.	No	NA	Spont. Remission
11	r(20)	Ring chromosome 20	Augustijn, 2001	4	NCSE	11 y, 14 y, 11y, 8y	12 y, 14 y, 11.5 y, 8.3 y	Gen. epi activity, F‐predom	Mental slowing	No	NA	NA
12	r(20)	Ring chromosome 20	Shirasaka, 2002	1	AASE	15 y	18 y	Gen. epi activity, F‐predom	Drooling, ic	No	DZP	Resolved
13	r(20)	Ring chromosome 20	Locharernkul, 2005	2	CPSE	NA	25 y, 37 y	Prol. gen. rhyth. 3‐5 Hz ShW or SlW, few S	Fluctuating consciousness, seldom rare my	In some occasions verbal stimuli	PT 1: PHT, VPA; PT 2: VPA	Spont. remission (temp. effect of ASM on EEG)
14	r(20)	Ring chromosome 20	Zou, 2006	1	NCSE	NA	26 y	NA	NA	NA	NA	NA
15	r(20)	Ring chromosome 20	Elghezal, 2007	1	NCSE	NA	12 y	Gen ↑‐volt. Θ (4‐5 Hz), occasional SW	Confusional states	NA	NA	NA
16	r(20)	Ring Chromosome 20	Alpman, 2005	1	AASE or NCSE	NA	NA	NA	Prolonged absence	NA	NA	NA
17	r(20)	Ring chromosome 20	Jacobs, 2008	1	NCSE	13 y	13 y	Cont. gen 2.5Hz SW, max. F	Prolonged absence	NA	Propofol, PB, MDZ, Thiopental Pentobarbital	Super‐refractory, lethal
18	r(20)	Ring Chromosome 20	Vignoli, 2009	3	NCSE	NA	NA, NA, 20 y	Cont, gen, bi‐F‐predom. Sl‐SW	Cognitive deterioration; aphasia in 1 pt; ic and slow automatic mov pt nr 3	NA	VPA+LTG	Refract., no recurrence after VPA+LTG
19	r(20)	Ring chromosome 20	Elens, 2012	6	NCSE	NA	4 y, 8 y, 53 y, 66 y, 22 y, 19 y	NA	Behav arrest and ic	NA	NA	NA
20	r(20)	Ring chromosome 20	Radhakrishnan, 2012	2	NCSE/CPSE	NA	15 y, 20 y	Gen, rhyth., medium ampl. Θ ‐‐> 1.5–3 Hz gen Sl‐SW	Clouding of consciousness	NA	NA	Spont. remission
21	r(20)	Ring chromosome 20	Vignoli, 2016	22	NCSE	NA	NA	Cont. gen SW, F‐predom	NA	NA	NA	Spont. remission
22	r(20)	Ring chromosome 20	Bayat, 2022	1	AASE	NA	NA	NA	Ic and motor slowdown	NA	NA	NA
23	r(17)	Ring chromosome 17	Coppola, 2018	1	NCSE	28 y	28 y	Subcont. gen SW	no behav. change	NA	BDZ	Improv. after treatment
24	r(17)	Ring chromosome 17	Ricard‐Mousnier, 2007	1	diurnal ESE	3 y	4 y	Ge Sl‐SW central‐predom (0,5–3 Hz); hours	Ic and motor slowdown	NA	NA	Spont. remission
25	4p‐	4‐p syndrome	Battaglia, 2003	1	AASE	2y6mo	NA	NA	Ic and motor slowdown	NA	NA	NA
26	4p‐	4‐p syndrome	Valente, 2003	1	AASE	30 mo	6 y	NA	Ic and motor slowdown	NA	NA	Not refractory
27	CNKSR2	CNKSR2‐DEE	Bonardi, 2020	2	ASE	7y6mo,8 y	11y, 8y	NA	Ic, fluctuating E‐My and distal limbs	NA	NA	NA prolonged
28	KCNH2	KCNH2‐DEE	Ghimire, 2022	1	Absence epilepsy with Status Epilepticus	11 y	11 y	occasional, gen S and poly‐SW	NA	NA	ESM and ZNS	Resolved
29	NEXMIF	NEXMIF‐DEE	Ogasawara, 2020	1	NCSE	38 y	38 y	Gen S, poliS, ShW	Ic, staring, occasional E‐My	NA	NA	NA
30	NEXMIF	NEXMIF‐DEE	Wu, 2020	1	AASE	26 y	29 y	1.5–2.5 Hz semi‐rhyth. gen. SW; eye‐closure sensitivity	↓ responsiveness, mydriasis, E‐My	NA	MDZ	Resolved with MDZ
31	NEXMIF	NEXMIF‐DEE	Cioclu, 2021	1	NCSE	9 y	28 y	Cont., gen. S, poly‐SW, eye closure‐sensitivity; worse with HV and IPS	Ic	NA	LZP*	Resolved with LZP, recurrent
32	TRPM3	TRPM3‐DEE	Kang, 2021	1	AASE	7y2mo	7y2mo	NA	NA	NA	NA	NA
33	SYNGAP1	SYNGAP1‐DEE	Lo Barco, 2021	1	AASE	NA	NA	Eye‐closure sensitivity and FOS	Ic	NA	NA	NA
34	GABRB1	DEE45	Monfrini, 2023	1	AASE	NA	14 y	Prolonged 2 Hz SW	NA	Respiratory infection	NA	NA

**Abbreviations:** ↑‐volt, high voltage; ↓, reduced; AA, atypical absence; AASE, atypical absence status epilepticus; ampl, amplitude; BDZ, benzodiazepines; behav, behavioral; CNZ, Clonazepam; cont., continuous; dev, deviation; diff, diffuse; DZP, Diazepam; E‐My, eyelid myoclonia; epi, epileptic; ESM, Ethosuximide; ext, extension; F, frontal; FOS, fixation‐off sensitivity; freq, frequent; gen, generalized; HV, hyperventilation; hypersaliv, hypersalivation; ic, impaired consciousness; imp, impaired; improv, improvement; IPS, intermittent photic stimulation; irreg, irregular; IV, intravenous; KD, ketogenic diet; LTG, Lamotrigine; LZP, Lorazepam; max, maximum; MDZ, Midazolam; mo, months; mov, movements; My, myoclonic; NA, not available; NCSE, Non convulsive status epilepticus; Nr, number; PB, Phenobarbital; PHT, Phenytoin; pred, predominant; pts, patients; refract, refractory, resp, responsive; rhyth, rhythmic; S, spike; Sl, slow; SlW, slow wave; spont, sponateneous; stereotyp, stereotypies; subcont, subcontinous; subseq, subsequent; SW, spike‐wave; sz, seizure; T, tonic; temp, temporary; TRH, Thyrotropin‐releasing hormone; UL, upper limbs; up, upward; VPA, valproic acid; y, years; ZNS, Zonisamide; α, alfa; θ, theta.

**FIGURE 2:**
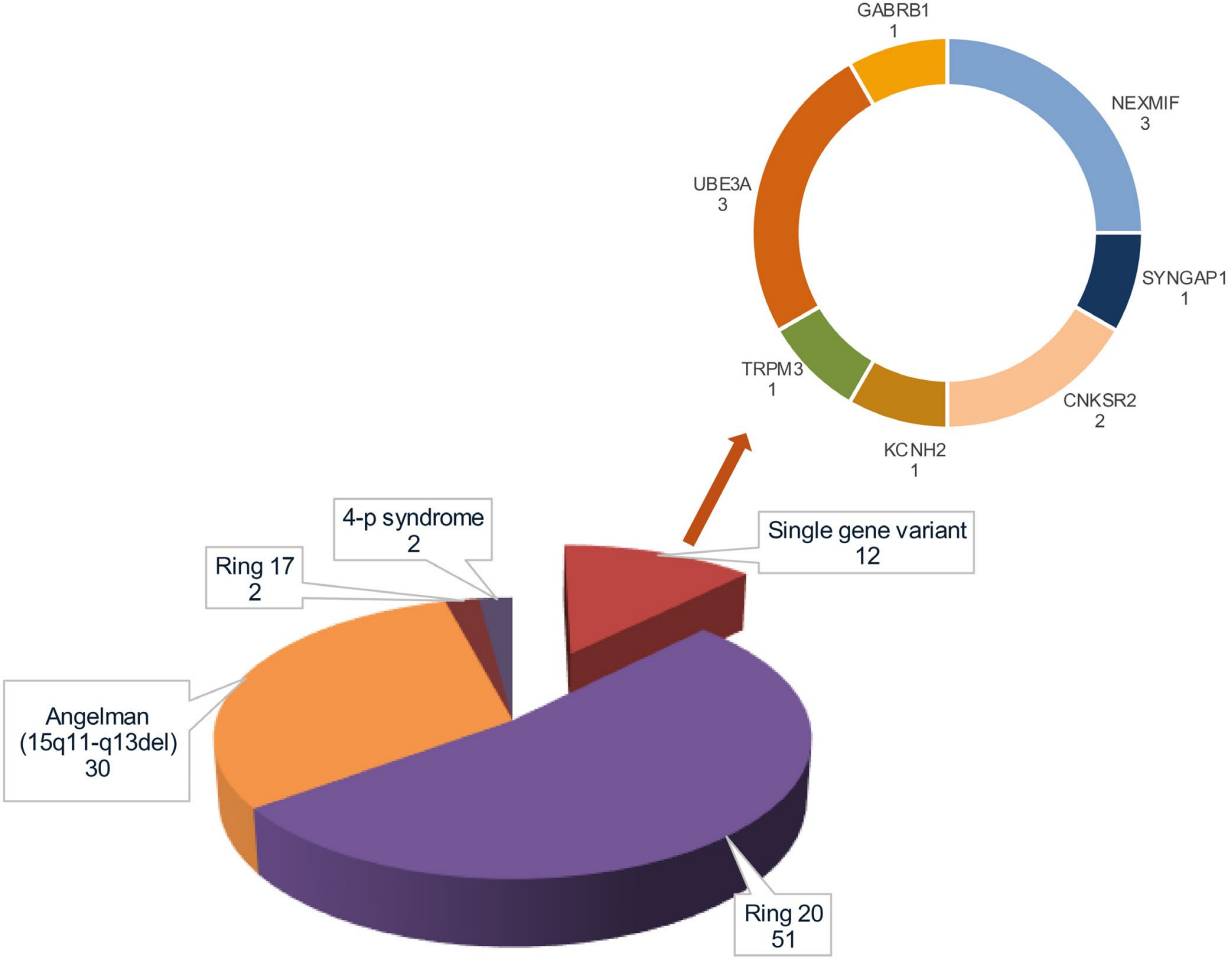
Distribution of genetic diagnoses in patients with reported AASE. The main pie chart shows the distribution of genetic diagnoses. The inset pie chart summarizes single‐gene etiologies; for each condition, the number reported indicates the number of patients.

These involve chromosomal abnormalities (24/34 articles)^14^, ^15^, ^16^, ^17^, ^19^, ^20^, ^22^, ^23^, ^24^, ^25^, ^26^, ^27^, ^28^, ^29^, ^30^, ^31^, ^32^, ^33^, ^34^, ^35^, ^36^, ^37^, ^38^, ^39^ and single‐gene variants (10/34 articles).^18^, ^21^, ^40^, ^41^, ^42^, ^43^, ^44^, ^45^, ^46^, ^47^ Overall, 97 patients with AASE have been described, of whom 85 (88%)^14^, ^15^, ^16^, ^17^, ^19^, ^20^, ^22^, ^23^, ^24^, ^25^, ^26^, ^27^, ^28^, ^29^, ^30^, ^31^, ^32^, ^33^, ^34^, ^35^, ^36^, ^37^, ^38^, ^39^ presented with a structural chromosomal abnormality, whereas 12 (12%) had a single‐gene variant, with seven different genes identified (*UBE3A*, *CNKSR2*, *KCNH2*, *NEXMIF*, *TRPM3*, *SYNGAP1*, *GABRB1*) (Figure 2).^18^, ^21^, ^40^, ^41^, ^42^, ^43^, ^44^, ^45^, ^46^, ^47^ For four of these genes, we could find only one patient in whom this association was found.^42^, ^45^, ^46^, ^47^

Below is a summary of findings across the different genetic conditions in which AASE was identified. Key features of AASE for each diagnosis are summarized in Table 2.

**TABLE 2:** Summary of the main findings in the different genetic conditions. For each genetic diagnosis, we provide a concise overview of the key clinical features and AASE‐related findings. The denominator indicates the number of patients for whom the information was available.

Condition (gene/Chrom/syndrome)	Age at AASE (median if *n* > 3)	Semiology	Triggers	Treatment	Outcome SR/TR/REF	EEG AASE	Sz types (main)	Other SE types	Drug resistant (yes)	ID/DD (yes)	ID/DD degree (mild/mod/sev/profound)	Notes
AS	3 y 7 mo	Imp responsiv.	PHT (1/33), CBZ (2/33), fever (1/33)	BDZ, TRH	SR 1/7, TR 5/7, REF 2/7	Diffuse, cont., SW bursts (1.5–3 Hz)	My, AA, At	GTCSE, NCSE	9/11	20/20	Sev	Freq inappropr laughter
Ring 20	11 y 6 mo	Clouding consciousness, confusion	HV (1/51), verbal stimuli (1/51)	VPA, BDZ	SR 29/37, TR 4/37, REF 4/37	SlW/ShW, slow SW (1.5–4.5 Hz), diffuse, F‐predom	FIC, TC, AA	NCSE	48/50	37/44	Mild/mod (32/34)	Often cognitive or behav problems after sz onset
Ring 17	3 y and 28 y	Imp responsiv. motor slowdown	—	BDZ (1 pt)	SR 1/2, TR 1/2	Subcont/cont gen Sl‐SW/SW; central‐predom (0.5–3 Hz) in 1 pt	GTC, T, C	No	2/2	2/2	Mod	—
4p syndrome	30 mo (1 pt)	Ic	NA	NA	NA	NA	GTC, My, AA	MySE (1/2)	1/2	2/2	Sev	—
CNKSR2	7 y 6 mo and 8 y	Ic	NA	NA	NA	NA	My, AA, GTC	SWAS	1/2	2/2	Mod‐Sev	regr., stereotypies, speech and facial dyspraxia, HA
KCNH2	11 y	NA	NA	ESM and ZNS	TR (1/1)	Occasional, gen S and poly‐SW	AA	No	No	1/1	Mild	ASD, ADHD
NEXMIF	38 y, 26 y, 9 y	ic, e‐my	NA	BDZ	TR (2/2)	Cont., gen. S, poly‐SW (1.5–2.5 Hz); eye closure‐sensitivity	AA, GTC	No	2/3	3/3	Mild	—
TRPM3	7 y 2 mo (1 pt)	NA	NA	NA	NA	NA	Sp/T	No	1/1	1/1	Sev	Mild hypo, dysm feat.
SYNGAP1	NA	Ic	NA	NA	NA	Eye closure sensitivity and FOS	My, At, AAAt, T	NA	1/1	1/1	Sev	—
GABRB1	NA	Ic	Resp infections	NA	NA	Prolonged 2 Hz SW	Fo, Fo migr, T	No	1/1	1/1	Profound	Acquired ↓HC, tetraparesis, dysphagia, restrictive pulmonary syndrome, OSAS

Abbreviations: AA, atypical absence; AAAt, atypical absence with atonic features; AASE, atypical absence status epilepticus; ADHD, attention deficit hyperactivity disorder; AS, Angelman syndrome; ASD, autistic spectrum disorder; At, atonic; BDZ, benzodiazepines; behav, behavioral; chrom, chromosomal abnormality; cont., continuous; DD, developmental delay; dysm, dysmorphic; E‐My, eyelid myoclonia; epi, epileptic; ESM, Ethosuximide; F, frontal; FIC, focal seizures with impaired consciusness; Fo, focal; FOS, fixation‐off sensitivity; freq, frequent; gen, generalized; GTC, generalized tonic–clonic; GTCSE, generalized tonic–clonic status epilepticus; HA, hyperactivity; HV, hyperventilation; hypo, hypotonia; ic, impaired consciousness; ID, intellectual disability; imp, impaired; inappr, inappropriate; MDZ, midazolam; migr, migrating; mo, months; mod, moderate; My, myoclonic; MySE, myoclonic status epilepticus; NA, not available; NCSE, Nonconvulsive status epilepticus; Nr, number; OSAS, obstructive sleep apnea syndrome; PHT, phenytoin; pred, predominant; ref., refractory; regr, regression; S, spike; SE, status epilepticus; Sev, severe; Sl, slow; SlW, slow wave; sp., spasms; SR, spontaneous remission; subcont, subcontinuous; SW, spike–wave; sz, seizure; T, tonic; TR, treatment‐responsive; TRH, thyrotropin‐releasing hormone; VPA, valproic acid; y, years; ZNS, zonisamide; ↓HC, reduced head circumference (microcephaly).

### 3.1 Angelman syndrome

Thirty‐three patients with Angelman syndrome and AASE were reported in eight articles.^14^, ^15^, ^16^, ^17^, ^18^, ^19^, ^20^, ^21^ A 15q11–13 deletion was identified in 30/33 (91%) patients,^14^, ^15^, ^16^, ^17^, ^19^, ^20^ whereas a pathogenic variant in *UBE3A* gene was found in 3/33 (9%).^18^, ^21^

All patients were classified as having AASE except for the patient described by Sugimoto et al., in whom the episode was defined as a “form of nonconvulsive generalized status epilepticus.”^15^

Among patients for whom the information was available (10/33),^14^, ^15^, ^17^, ^18^, ^21^ the age of onset of status epilepticus ranged between 5 months and 19 years of age (median 43.5 months).

EEG findings usually showed continuous, generalized epileptiform activity, at a frequency of 1.5–2.5 Hz^14^, ^15^, ^21^; a different picture has been described by Espay et al., who report a patient with a pathogenic variant in *UBE3A*, who presented with repeated atypical absences without return to baseline conditions between seizures and an EEG characterized by bursts of frontally predominant rhythmic alpha activity.^18^

Clinically, AASE was reported as characterized by impaired consciousness in four studies,^14^, ^15^, ^20^, ^21^ with subtle changes in mood/behavior in two papers,^18^, ^21^ associated with eyelid myoclonia in two.^14^, ^18^

Information regarding SE treatment and outcome was available for seven patients: in 4/7, the status resolved or markedly improved after benzodiazepine treatment, 1/7 was defined as nonresponsive to diazepam, 1/7 recovered spontaneously, while 1/7 required treatment with ketogenic diet.^14^, ^15^, ^18^, ^21^ ICU admission and anesthetic treatment were not required in any of the reported cases.

Other types of SE reported in these patients were: convulsive status epilepticus (CSE), tonic status epilepticus, and myoclonic status epilepticus.^16^, ^17^, ^19^, ^20^

### 3.2 Ring chromosome 20

We found 14 articles describing 51 patients with electroclinical features of AASE.^22^, ^23^, ^24^, ^25^, ^26^, ^27^, ^28^, ^29^, ^30^, ^31^, ^32^, ^33^, ^34^, ^35^

Most authors defined this condition as NCSE (6/14),^24^, ^27^, ^29^, ^30^, ^32^, ^34^ in four cases it was defined as AASE,^23^, ^25^, ^28^, ^35^ while in three papers, it was outlined as CPSE,^22^, ^26^, ^33^ based on evidence suggesting a focal origin of seizures in the frontal lobes, through EEG, MEG, and SPECT studies.^22^ Vignoli et al. (2009) refer to this condition both as AASE and NCSE, but they also support the hypothesis that ring chromosome 20 is a network syndrome, with a main involvement of frontal lobes—basal ganglia networks.^31^

The median age of onset of status epilepticus was 11 years (range 4–15 years, information available for 11/50 patients).^22^, ^23^, ^24^, ^25^, ^30^

Detailed EEG information was reported in 10/14 studies.^22^, ^23^, ^24^, ^25^, ^26^, ^29^, ^30^, ^31^, ^33^, ^34^ The EEG during status showed slow wave or sharp‐wave–/slow spike‐and‐wave activity, at frequencies ranging between 1.5 and 4.5 Hz, diffuse but predominant over the frontal regions.^22^, ^23^, ^24^, ^25^, ^26^, ^29^, ^30^, ^31^, ^33^, ^34^ The clinical features were generally characterized by motor slowdown, clouding of consciousness, frightened expression, and in some cases automatisms.

Information regarding SE episodes outcome was available in 7/14 papers^22^, ^25^, ^26^, ^30^, ^31^, ^33^, ^34^: In two patients, SE was reported as refractory or superrefractory,^30^, ^31^ while in three, it underwent a spontaneous remission^26^, ^33^, ^34^; Inoue et al. report a positive response to diazepam in one patient and lidocaine in another patient, while no information is available for treatment response during status in the remaining four patients;^22^ diazepam terminated the episodes of AASE also in the patient described by Shirasaka.^25^ One patient required anesthetic treatment,^30^ yet status epilepticus was fatal in this case.

### 3.3 Ring chromosome 17

Two patients with ring chromosome 17 and AASE were found.^36^, ^37^ The authors defined this condition as NCSE in one case^37^ and diurnal electrical status epilepticus in the other.^36^

The age at status epilepticus was 3 years in one^36^ and 28 years in the other.^37^ The status in the patient described by Coppola et al.,^37^ was characterized by impaired alertness and motor slowdown; the EEG showed subcontinuous generalized spike‐and‐wave activities. The video‐EEG recording of the patient described by Ricard‐Mousnier et al. showed continuous epileptic activity (spike‐and‐wave), with a frequency of 0.5–3 Hz, diffuse but predominant on the central regions of both hemispheres.^36^

The patient described by Coppola et al.^37^ was treated with benzodiazepines with a favorable response,^37^ whereas the patient reported by Ricard‐Mousnier et al. underwent spontaneous remission.^36^ Neither of the two reported patients required anesthetic treatment.

### 3.4 4p syndrome—Wolf–Hirschhorn syndrome (WHS)

Two patients with AASE and a partial deletion of the short arm of chromosome 4 have been reported in two different articles, and in both cases, it was defined as AASE.^38^, ^39^ The age of onset was reported in one of the two patients^39^ and was 30 months; from a clinical point of view, she presented with recurrent atypical absences, lasting for days with impaired consciousness; the status in this patient was described as not refractory to treatment.^39^ Battaglia et al.^38^ describe six patients with 4p syndrome, including one patient with AASE. He presented with multiple seizure types, and his epilepsy was defined as refractory to treatment without further details. His ictal EEG showed brief, diffuse bursts of rhythmic slow waves (2–3 Hz), with superimposed spikes.

### 3.5 Monogenic variants

Excluding the three patients with a variant in the gene *UBE3A*, who were discussed in the Angelman syndrome section,^18^, ^21^ nine additional patients were reported in eight different articles as having a pathogenic variant in a single gene and a clinical and EEG picture consistent with AASE.^40^, ^41^, ^42^, ^43^, ^44^, ^45^, ^46^, ^47^

The involved genes are *CNKSR2* (two patients),^40^ *NEXMIF* (three patients),^41^, ^42^, ^43^ *TRPM3* (one patient),^44^ *SYNGAP1* (one patient),^45^ *KCNH2* (one patient),^46^ and *GABRB1* (one patient).^47^

The median age at first status occurrence in these patients was 9 years (range 7 years and 2 months – 38 years, information available in 7/9 patients).^40^, ^41^, ^42^, ^43^, ^44^, ^46^

EEG during status in patients with *NEXMIF* variants,^41^, ^42^, ^43^ defined as NCSE, showed continuous generalized spikes, polyspikes, and sharp waves. In one patient,^43^ eye closure sensitivity and worsening with hyperventilation and intermittent photic stimulation were reported. The patient with a *KCNH2* pathogenic variant^46^ presented clinically with altered mental status, eyelid fluttering, and urinary incontinence; occasional bursts of 1 s, diffuse spikes, and poly‐spike‐and‐wave discharges were observed on the EEG, and the diagnosis was “non‐intractable absence epilepsy with status epilepticus.”

No detailed information regarding EEG during AASE was presented in the patients with *CNKSR2* variants, the authors defined the condition as “absence status epilepticus.”^40^

The condition was instead defined as AASE in the patient with the *TRPM3* variant.^44^

The patient with the *GABRB1* variant presented episodes of AASE, often triggered by respiratory infections, in late childhood. During these episodes, EEG recordings showed prolonged 2 Hz spike‐and‐wave discharges. The coexistence of these episodes with tonic seizures was consistent with Lennox–Gastaut syndrome.^47^

Regarding SE outcome, in the five patients in whom the information was reported,^40^, ^42^, ^43^, ^46^ the AASE resolved with first‐ or second‐line treatment in 3/5^42^, ^43^, ^46^ while in the remaining two patients,^40^ AASE was prolonged but no information regarding treatment was available.

## 4 DISCUSSION

Until now, not many studies have analyzed the prevalence of status epilepticus in genetic forms of epilepsy. However, a recent study showed that in a cohort of 510 patients with a DEE related to pathogenic variants (*SCN1A*, *SCN2A*, *SCN8A*, *SYNGAP1*, *NEXMIF*, *CHD2*, *PCDH19*, *STXBP1*, *GRIN2A*, *KCNT1*, *KCNQ2*), NCSE was less frequent than CSE and was described in 19% of cases (vs. 47% of patients with CSE).^48^ NCSE was mainly reported in non‐Dravet patients with pathogenic variants in *SCN1A*, in patients with Angelman syndrome, and in patients with variants in *CHD2* and *NEXMIF*.^48^

The aim of our study was to describe the genetic conditions in which AASE, a rare subtype of NCSE, has been reported, and to explore the potential diagnostic relevance of this finding for genetic diagnosis. We concurrently reviewed treatments and outcomes in cases where these data were available.

We identified 97 patients with a clinical‐electrographic picture consistent with AASE and a diagnosed genetic etiology. Most of them (88%) had a chromosomal abnormality, whereas 12 patients showed a single‐gene variant.

Age at first reported AASE was highly variable, from early infancy (5 months in the patient with Angelman syndrome reported by Valente et al.^20^), through childhood–adolescence and into adulthood (38 years in a patient with a *NEXMIF* pathogenic variant).^41^ Most reports focused on an index episode, lacking a longitudinal follow‐up; therefore, in most cases, the age of onset and the age at last observed AASE were the same (Table 1). Notably, in patients with ring chromosome 20 in whom this information was available, there was a significant gap between the first and the last observed episodes, confirming the known tendency of recurrence of status epilepticus in these patients. In the published cases, an earlier onset seemed to be reported in Angelman syndrome, whereas a later presentation was more commonly reported in patients with ring chromosome 20 and *NEXMIF* pathogenic variants. However, this observation should be interpreted with caution, since age data were frequently missing. Moreover, the small number of cases for many etiologies limits the ability to draw definitive conclusions. Nonetheless, based on the available reports, a later age at presentation of AASE should not preclude consideration of a genetic etiology and appropriate genetic testing.

### 4.1 AASE in the context of chromosomal abnormalities

The conditions most frequently associated with AASE were ring chromosome 20 (51 patients) and Angelman syndrome caused by a 15q11–q13 deletion (30 patients); however, especially for the former, there was a lack of agreement between different authors in the classification of the type of NCSE observed.

AASE was mainly described in patients with a high seizure burden with many different seizure types and often drug resistance; most of them were reported having intellectual disability (reported in 21/23 of the articles in which the information was available).

In many cases, AASE was not the only type of SE reported: patients with Angelman syndrome have been reported having convulsive SE, myoclonic SE, and tonic SE,^16^, ^17^, ^19^, ^20^ while in one patient with 4‐p syndrome, myoclonic status epilepticus was described.^39^

In the majority of published cases with available outcome data, AASE was not reported as severe, although in two cases with ring chromosome 20^30^, ^31^ it was described as refractory or superrefractory, one resulting in a fatal outcome.^30^ In the remaining articles in which there was some information available (13/26), NCSE either underwent spontaneous remission (6/13)^18^, ^23^, ^26^, ^33^, ^34^, ^36^ or it responded to first or second‐line treatments (6/13).^14^, ^15^, ^21^, ^22^, ^25^, ^37^ In the patient with the 4p‐syndrome, described by Valente et al., status was described as “not refractory.”^39^

These considerations might suggest that, in many cases, aggressive treatment may not be necessary. In line with this, Shirasaka^25^ did not find increased serum NSE levels in these patients and hypothesized a lack of neuronal damage. However, this interpretation should be regarded with caution given the nature of the available evidence. Moreover, a different perspective might derive from reports of patients with ring chromosome 20, where a close temporal relation has been described between the onset of seizures and SE and the development of cognitive impairment.^34^, ^49^ However, seizure activity appears to be only one contributing factor in a more complex developmental scenario, since cognitive outcomes appear also to correlate with the age at the onset of seizures^39^ and the proportion of ring‐20 mosaicism.^50^

### 4.2 AASE in the context of monogenic variants

As with regards to monogenic forms of epilepsy, AASE was described in association with pathogenic variants in seven genes: *UBE3A* (three patients with Angelman syndrome)^18^, ^21^ *CNKSR2*, two patients,^40^ *NEXMIF*, three patients,^41^, ^42^, ^43^ *TRPM3*, one patient,^44^ *SYNGAP1*, one patient,^45^ *KCNH2*, one patient,^46^ and *GABRB1*, one patient.^47^ Three of these genes encode for ion channels: *KCNH2*, which encodes for the alpha subunit of a voltage‐gated potassium channel,^46^ *TRPM3*, which encodes for a nonselective cation channel,^44^ and *GABRB1*, which encodes the beta‐1 subunit of the gamma‐aminobutyric acid (GABA) type A receptor.^47^ *CNKSR2*, *NEXMIF*, and *SYNGAP1* are genes involved in the development and regulation of synapses and synaptic plasticity, and two of them (*CNKSR2* and *NEXMIF*) are located on the X chromosome.^51^, ^52^, ^53^ *UBE3A* is involved in ubiquitin‐mediated protein degradation, maintenance of adequate GABA levels, and regulation of synaptic development and plasticity.^54^ *GABRB1* is involved in mediating inhibitory neurotransmission; *TRPM3* is implicated in neuronal excitability through Ca^2+^ influx, while *KCNH2* regulates membrane excitability.^55^, ^56^, ^57^

A recent multicenter study investigated patients with absence seizures and a monogenic cause of epilepsy. Interestingly, among the genes found in the 160 patients included, only *NEXMIF* and *SYNGAP1* overlapped with the AASE‐genes we found in literature. No patients harboring pathogenic variants in *UBE3A*, *CNKSR2*, *TRPM3*, *GABRB1*, and *KCNH2* have been reported in this study.^58^ Similarly, no pathogenic variants in these genes have been reported as associated with atypical absences in a recent review on the topic.^59^ As discussed also by Bhatnagar and Shorvon,^60^ this discrepancy might suggest that the mechanisms leading to status epilepticus are different from the ones sustaining seizures. Absence seizures appear to result from a dysregulation within cortico‐thalamo‐cortical circuits^60^, ^61^ but without the alteration of the inhibitory mechanisms, allowing for their spontaneous termination; on the contrary, status epilepticus arises from the failure of seizure‐terminating mechanisms and the initiation of self‐maintaining excitatory mechanisms through dysfunctional synaptic plasticity processes.^3^, ^62^, ^63^ In this context, we could hypothesize that *UBE3A*, *GABRB1*, *CNKSR2*, *TRPM3*, and *KCNH2* may preferentially affect the inhibitory–excitatory balance and neuronal excitability and possibly influence the probability of transition to a post‐ictal state. However, this remains highly speculative given the small number of reported cases.

Notably, in most patients with AASE and a single‐gene variant, AASE did not appear to be a core clinical feature across variants; the main exception was *NEXMIF*, which also had the largest number of reported patients (three patients).^41^, ^42^, ^43^ NCSE is not infrequent in these patients; in fact, in a cohort of 87 patients with *NEXMIF* encephalopathy, NCSE was described in 13 patients, mainly with myoclonic and absence components.^52^ Two of the three patients with a *NEXMIF* gene variant and AASE,^42^, ^43^ shared a similar clinical and EEG picture: patients presented prolonged episodes of psychomotor slowdown/impaired consciousness, confusion and fluttering of eyelids, and EEG showed generalized epileptic activity, with eye closure sensitivity.

## 5 STUDY LIMITATIONS

This review presents some limitations; first, because of the heterogeneous definitions, we have included patients not overtly classified as AASE by the authors, by relying on available EEG and clinical data reported in the articles. At the same time, we have also included patients that were classified by the authors as having AASE, in some cases without the possibility to review EEG and clinical data that led to the definition. We have also included patients with ring chromosome 20 syndrome despite the evidence from advanced neuroimaging studies of a frontal lobe generator with the involvement of basal ganglia—prefrontal networks.^22^, ^49^ If, on the basis of these observations, cases with ring chromosome 20 syndrome were to be excluded from the analysis and classified as focal NCSE, as argued by other authors, the proportion of patients with AASE associated with a chromosomal abnormality would decrease to 74%. However, for the purposes of this review, we adopted a more practical and clinically oriented approach, focusing on the electroclinical phenotype encountered in routine clinical practice.

For similar reasons, we suspect that AASE might be underrecognized and underreported. Both the clinical features and the EEG changes associated with it are often not striking, consisting only in a slight worsening of the baseline conditions, therefore making this condition not easily recognized and diagnosed. Moreover, despite updated classifications and a widespread effort toward standardizing the terminology used, there is not always a perfect agreement among different authors.

This could be particularly true in large cohort studies, where there may be limited information about the clinical and EEG data of patients classified as having NCSE. Therefore, deeper phenotyping and more extensive details regarding the type of status epilepticus and EEG in these patients could lead to a better understanding of the real prevalence of this condition. In this regard, also Donnan et al.^48^ report a possible underestimation of NCSE in DEEs with subtle, frequent nonconvulsive seizure types, such as *SYNGAP1*, where patients present almost continuous absence seizures with eyelid myoclonias associated with epileptiform activity on EEG; therefore, this appears to be especially relevant for patients with AASE.

Moreover, because terminology and diagnostic criteria are not uniform, we cannot exclude incomplete retrieval of relevant cases. This could result both in an underestimation of genetic AASE and an overrepresentation of etiologies in which AASE is more consistently recognized and classified as such.

Finally, we could not systematically assess genotype–phenotype concordance. Therefore, it is not possible to assess whether individuals with atypical presentations are more likely to present with AASE compared with those with more classical syndromic features.

## 6 PRACTICAL IMPLICATIONS AND FUTURE PERSPECTIVE

**FIGURE 3:**
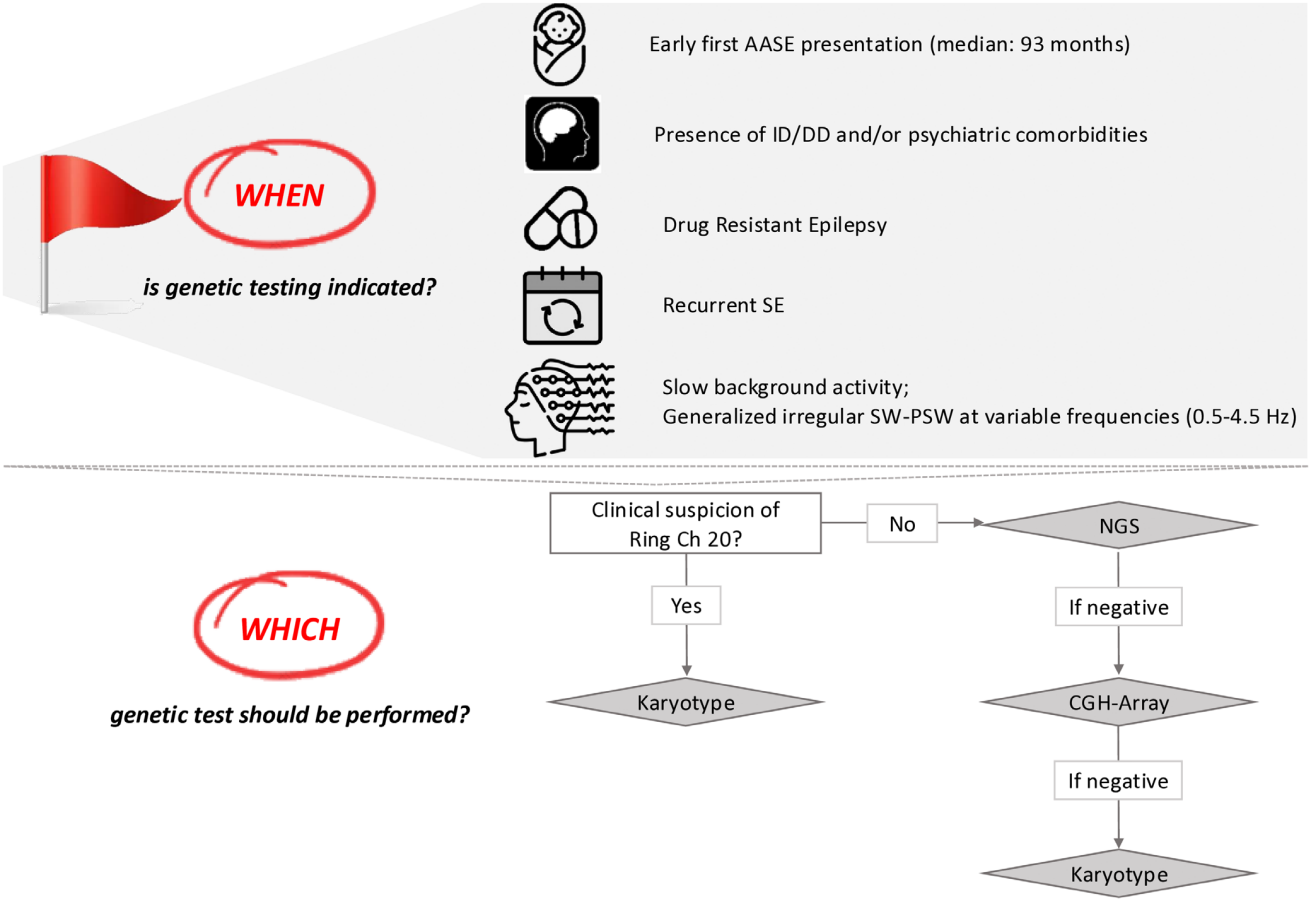
When to test and Which Genetic Tests to Perform in AASE. Suggested decision framework for genetic testing in patients with AASE. Top panel: Clinical and EEG red flags supporting genetic testing. Lower panel: An illustrative testing workflow.

Bearing in mind the probable underrecognition of AASE in monogenic forms of DEEs, as well as potential publication bias, our findings suggest considering chromosomal abnormalities in patients with a suspected genetic etiology and this clinical and electrographic picture (see Figure 3).

Moreover, this review highlights the importance of accurate phenotyping and use of the appropriate classification to gain more insight into the clinical picture of the patients and to guide diagnosis as well as to improve the generalizability of research findings. As a future perspective, a multicenter study collecting patients with AASE and a genetic cause of epilepsy, as recently performed for monogenic absence seizures,^57^ could help in better clarifying the genetic landscape of the epilepsies in which this electroclinical pattern occurs.

## 7 CONCLUSION

We reviewed the available literature on genetic etiologies of epilepsies in which AASE has been reported. In the published cases we identified, this condition appears to be more frequently described in individuals with chromosomal abnormalities. However, this observation should be interpreted with caution since it may reflect reporting and recognition biases rather than a true etiologic distribution pattern. In particular, AASE may be more readily recognized and reported in syndromes where it is already considered part of the expected phenotype, while it may be underrecognized or described using less specific terminology in other genetic conditions. Ring chromosome 20 syndrome and Angelman syndrome have distinctive clinical features that make them readily recognizable in epilepsy centers, and their association with NCSE has long been established in literature; therefore, further reinforcing recognition and reporting of this pattern. Conversely, this may be less applicable to monogenic forms of DEEs, many of which have only been characterized more recently.

More detailed phenotyping and a more standardized use of terminology could improve diagnostic accuracy and could enhance comparability across studies. Future multicentric studies and registries applying harmonized AASE definitions and comprehensive reporting of genetics, EEG features, treatments, and outcomes would be necessary to better define prevalence, delineate etiologic associations, and potentially identify predictors of treatment response.

## AUTHOR CONTRIBUTIONS

Maria Cristina Cioclu and Giada Giovannini independently assessed studies for inclusion. Any disagreements were resolved through discussion with Stefano Meletti. All three authors contributed to the conception and design of the review, interpretation of data, and drafting and critical revision of the manuscript. All authors approved the final version of the manuscript.

## CONFLICT OF INTEREST STATEMENT

The authors declare no conflicts of interest.

## ETHICS STATEMENT

We confirm that we have read the journal's position on issues involved in ethical publication and affirm that this article is consistent with those guidelines.

## Supporting information


Data S1

## Data Availability

No new data were created or analyzed in this study. This review article is based on data from published articles.

## References

[1] Sutter R , Semmlack S , Kaplan PW . Nonconvulsive status epilepticus in adults: insights into the invisible. Nat Rev Neurol. 2016;12(5):281–293. 10.1038/nrneurol.2016.45 27063108

[2] Kinney MO , Craig JJ , Kaplan PW . Hidden in plain sight: non‐convulsive status epilepticus—recognition and management. Acta Neurol Scand. 2017;136(4):280–292. 10.1111/ane.12732 28144933

[3] Trinka E , Cock H , Hesdorffer D , Rossetti AO , Scheffer IE , Shinnar S , et al. A definition and classification of status epilepticus: report of the ILAE task force on classification of status epilepticus. Epilepsia. 2015;56(10):1515–1523. 10.1111/epi.13121 26336950

[4] Striano P , Minassian BA . From genetic testing to precision medicine in epilepsy. Neurotherapeutics. 2020;17(2):609–615. 10.1007/s13311-020-00835-4 31981099 PMC7283411

[5] Balestrini S , Mei D , Sisodiya SM , Guerrini R . Steps to improve precision medicine in epilepsy. Mol Diagn Ther. 2023;27(6):661–672. 10.1007/s40291‐023‐00676-9 37755653 PMC10590329

[6] Fernández‐Torre JL , Kaplan PW , Hernández‐Hernández MA . New understanding of nonconvulsive status epilepticus in adults: treatments and challenges. Expert Rev Neurother. 2015;15(12):1455–1473. 10.1586/14737175.2015.1115719 26559043

[7] Drislane FW , Kaplan PW , Herman ST . Nonconvulsive status epilepticus. In: Schomer DL , Lopes da Silva FH , editors. Niedermeyer's electroencephalography: basic principles, clinical applications, and related fields. 6th ed. Philadelphia: Lippincott Williams & Wilkins; 2011. p. 595–644.

[8] Koutroumanidis M . Absence status epilepticus. In: Kaplan PW , Drislane FW , editors. Nonconvulsive status epilepticus. New York: Demos Medical Publishing; 2009. p. 153–173.

[9] Hamad AP , Ferrari‐Marinho T , Caboclo LO , Thomé U , Fernandes RMF . Nonconvulsive status epilepticus in epileptic encephalopathies in childhood. Seizure. 2020;80:212–220. 10.1016/j.seizure.2020.06.024 32645639

[10] Leitinger M , Beniczky S , Rohracher A , Gardella E , Kalss G , Qerama E , et al. Salzburg consensus criteria for non‐convulsive status epilepticus: approach to clinical application. Epilepsy Behav. 2015;49:158–163. 10.1016/j.yebeh.2015.05.007 26092326

[11] Page MJ , McKenzie JE , Bossuyt PM , Boutron I , Hoffmann TC , Mulrow CD , et al. The PRISMA 2020 statement: an updated guideline for reporting systematic reviews. BMJ. 2021;372:n71. 10.1136/bmj.n71 33782057 PMC8005924

[12] Campbell M , McKenzie JE , Sowden A , Katikireddi SV , Brennan SE , Ellis S , et al. Synthesis without meta‐analysis (SWiM) in systematic reviews: reporting guideline. BMJ. 2020;368:l6890. 10.1136/bmj.l6890 31948937 PMC7190266

[13] Ouzzani M , Hammady H , Fedorowicz Z , Elmagarmid A . Rayyan‐a web and mobile app for systematic reviews. Syst Rev. 2016;5(1):210. 10.1186/s13643-016-0384-4 27919275 PMC5139140

[14] Matsumoto A , Kumagai T , Miura K , Miyazaki S , Hayakawa C , Yamanaka T . Epilepsy in Angelman syndrome associated with chromosome 15q deletion. Epilepsia. 1992;33(6):1083–1090. 10.1111/j.1528-1157.1992.tb01763.x 1464268

[15] Sugimoto T , Yasuhara A , Ohta T , Nishida N , Saitoh T , Hamabe H , et al. Angelman syndrome in three siblings: characteristic epileptic seizures and EEG abnormalities. Epilepsia. 1992;33(6):1078–1082. 10.1111/j.1528-1157.1992.tb01762.x 1464267

[16] Laan LA , Renier WO , Arts WFM , Buntinx IM , vd Burgt JAM , Stroink H , et al. Evolution of epilepsy and EEG findings in Angelman syndrome. Epilepsia. 1997;38(2):195–199. 10.1111/j.1528-1157.1997.tb01097.x 9048672

[17] Minassian BA , Delorey TM , Olsen RW , Philippart M , Bronstein Y , Zhang Q , et al. Angelman syndrome: correlations between epilepsy phenotypes and genotypes. Ann Neurol. 1998;43(4):485–493. 10.1002/ana.410430412 9546330

[18] Espay AJ , Andrade DM , Wennberg RA , Lang AE . Atypical absences and recurrent absence status in an adult with Angelman syndrome due to the UBE3A mutation. Epileptic Disord. 2005;7(3):227–230. 16162432

[19] Uemura N , Matsumoto A , Nakamura M , Watanabe K , Negoro T , Kumagai T , et al. Evolution of seizures and electroencephalographical findings in 23 cases of deletion type Angelman syndrome. Brain Dev. 2005;27(5):383–388. 10.1016/j.braindev.2004.01.009 15963670

[20] Valente KD , Koiffmann CP , Fridman C , Varella M , Kok F , Andrade JQ , et al. Epilepsy in patients with Angelman syndrome caused by deletion of chromosome 15q11–13. Arch Neurol. 2006;63(1):122–128. 10.1001/archneur.63.1.122 16401744

[21] Melikishvili G , Bienvenu T , Tabatadze N , Gachechiladze T , Kurua E , Gverdtsiteli S , et al. Novel UBE3A pathogenic variant in a large Georgian family produces non‐convulsive status epilepticus responsive to ketogenic diet. Seizure. 2022;94:70–73. 10.1016/j.seizure.2021.11.012 34872019

[22] Inoue Y , Fujiwara T , Matsuda K , Kubota H , Tanaka M , Yagi K , et al. Ring chromosome 20 and nonconvulsive status epilepticus: a new epileptic syndrome. Brain. 1997;120(6):939–953. 10.1093/brain/120.6.939 9217679

[23] Petit J , Roubertie A , Inoue Y , Genton P . Non‐convulsive status in the ring chromosome 20 syndrome: a video illustration of three cases. Epileptic Disord. 1999;1(4):237–241. 10.1684/j.1950-6945.1999.tb00331.x 10937160

[24] Augustijn PB , Parra J , Wouters CH , Joosten P , Lindhout D , van Emde Boas W . Ring chromosome 20 epilepsy syndrome in children: electroclinical features. Neurology. 2001;57(6):1108–1111. 10.1212/WNL.57.6.1108 11571346

[25] Shirasaka Y . Lack of neuronal damage in atypical absence status epilepticus. Epilepsia. 2002;43(12):1498–1501. 10.1046/j.1528-1157.2002.10502.x 12460251

[26] Locharernkul C , Ebner A , Promchainant C . Ring chromosome 20 with nonconvulsive status epilepticus: electroclinical correlation of a rare epileptic syndrome. Clin EEG Neurosci. 2005;36(3):151–160. 10.1177/155005940503600305 16128150

[27] Zou YS , Van Dyke DL , Thorland EC , Chhabra HS , Michels VV , Keefe JG , et al. Mosaic ring 20 with no detectable deletion by FISH analysis: characteristic seizure disorder and literature review. Am J Med Genet A. 2006;140(16):1696–1706. 10.1002/ajmg.a.31332 16835934

[28] Alpman A , Serdaroglu G , Cogulu O , Tekgul H , Gokben S , Ozkinay F . Ring chromosome 20 syndrome with intractable epilepsy. Dev Med Child Neurol. 2007;47(5):343–346. 10.1111/j.1469-8749.2005.tb01146.x 15892377

[29] Elghezal H , Hannachi H , Mougou S , Kammoun H , Triki C , Saad A . Ring chromosome 20 syndrome without deletions of the subtelomeric and CHRNA4‐KCNQ2 gene loci. Eur J Med Genet. 2007;50(6):441–445. 10.1016/j.ejmg.2007.07.002 17851150

[30] Jacobs J , Bernard G , Andermann E , Dubeau F , Andermann F . Refractory and lethal status epilepticus in a patient with ring chromosome 20 syndrome. Epileptic Disord. 2008;10(4):254–259. 10.1684/epd.2008.0212 19017565

[31] Vignoli A , Canevini MP , Darra F , La Selva L , Fiorini E , Piazzini A , et al. Ring chromosome 20 syndrome: a link between epilepsy onset and neuropsychological impairment in three children. Epilepsia. 2009;50(11):2420–2427. 10.1111/j.1528-1167.2009.02176.x 19583784

[32] Elens I , Vanrykel K , De Waele L , Jansen K , Segeren M , Van Paesschen W , et al. Ring chromosome 20 syndrome: electroclinical description of six patients and review of the literature. Epilepsy Behav. 2012;23(4):409–414. 10.1016/j.yebeh.2012.02.008 22424860

[33] Radhakrishnan A , Menon RN , Hariharan S , Radhakrishnan K . The evolving electroclinical syndrome of epilepsy with ring chromosome 20. Seizure. 2012;21(2):92–97. 10.1016/j.seizure.2011.09.009 22000954

[34] Vignoli A , Bisulli F , Darra F , Mastrangelo M , Barba C , Giordano L , et al. Epilepsy in ring chromosome 20 syndrome. Epilepsy Res. 2016;128:83–93. 10.1016/j.eplepsyres.2016.10.004 27816898

[35] Bayat A , Fenger CD , Techlo TR , Højte AF , Nørgaard I , Hansen TF , et al. Impact of genetic testing on therapeutic decision‐making in childhood‐onset epilepsies: a study in a tertiary epilepsy center. Neurotherapeutics. 2022;19(4):1353–1367. 10.1007/s13311-022-01264-1 35723786 PMC9587146

[36] Ricard‐Mousnier B , N'Guyen S , Dubas F , Pouplard F , Guichet A . Ring chromosome 17 epilepsy may resemble that of ring chromosome 20 syndrome. Epileptic Disord. 2007;9(4):327–331. 10.1684/epd.2007.0121 17884758

[37] Coppola A , Morrogh D , Farrell F , Balestrini S , Hernandez‐Hernandez L , Krithika S , et al. Ring chromosome 17 not involving the miller‐Dieker region: a case with drug‐resistant epilepsy. Mol Syndromol. 2017;9(1):38–44. 10.1159/000479949 29456482 PMC5803681

[38] Battaglia D , Zampino G , Zollino M , Mariotti P , Acquafondata C , Lettori D , et al. Electroclinical patterns and evolution of epilepsy in the 4p‐ syndrome. Epilepsia. 2003;44(9):1183–1190. 10.1046/j.1528-1157.2003.63502.x 12919390

[39] Valente KD , Freitas A , Fiore LA , Kim CA . A study of EEG and epilepsy profile in wolf‐Hirschhorn syndrome and considerations regarding its correlation with other chromosomal disorders. Brain Dev. 2003;25(4):283–287. 10.1016/s0387-7604(02)00223-1 12767462

[40] Bonardi CM , Mignot C , Serratosa JM , Giraldez BG , Moretti R , Rudolf G , et al. Expanding the clinical and EEG spectrum of CNKSR2‐related encephalopathy with status epilepticus during slow sleep (ESES). Clin Neurophysiol. 2020;131(5):1030–1039. 10.1016/j.clinph.2020.01.020 32197126

[41] Ogasawara M , Nakagawa E , Takeshita E , Hamanaka K , Miyatake S , Matsumoto N , et al. Clonazepam as an effective treatment for epilepsy in a female patient with NEXMIF mutation: case report. Mol Syndromol. 2020;11(4):232–237. 10.1159/000510172 33224018 PMC7675231

[42] Wu D , Ji C , Chen Z , Wang K . Novel NEXMIF gene pathogenic variant in a female patient with refractory epilepsy and intellectual disability. Am J Med Genet A. 2020;182(11):2765–2772. 10.1002/ajmg.a.61848 32924309

[43] Cioclu MC , Coppola A , Tondelli M , Vaudano AE , Giovannini G , Krithika S , et al. Cortical and subcortical network dysfunction in a female patient with NEXMIF encephalopathy. Front Neurol. 2021;12:661447. 10.3389/fneur.2021.722664 PMC8459922 34566868

[44] Kang Q , Yang L , Liao H , Yang S , Kuang X , Ning Z , et al. A Chinese patient with developmental and epileptic encephalopathies (DEE) carrying a TRPM3 gene mutation: a paediatric case report. BMC Pediatr. 2021;21:256. 10.1186/s12887-021-02719-8 34074259 PMC8167971

[45] Lo Barco T , Kaminska A , Solazzi R , Cancés C , Barcia G , Chemaly N , et al. SYNGAP1‐DEE: a visual sensitive epilepsy. Clin Neurophysiol. 2021;132(4):841–850. 10.1016/j.clinph.2021.01.014 33639450

[46] Ghimire A , Banoub RW , Tobias JD . Anesthetic care of a child harboring the KCNH2 gene. J Med Cases. 2022;13(1):40–43. 10.14740/jmc3870 35211235 PMC8827253

[47] Monfrini E , Borellini L , Zirone E , Yahya V , Mauri E , Molisso MT , et al. GABRB1‐related early onset developmental and epileptic encephalopathy: clinical trajectory and novel de novo mutation. Epileptic Disord. 2023;25(6):867–873. 10.1002/epd2.20132 37518907

[48] Donnan AM , Schneider AL , Russ‐Hall S , Churilov L , Scheffer IE . Rates of status epilepticus and sudden unexplained death in epilepsy in people with genetic developmental and epileptic encephalopathies. Neurology. 2023;100(16):e1712–e1722. 10.1212/WNL.0000000000207080 36750385 PMC10115508

[49] Vaudano AE , Ruggieri A , Vignoli A , Avanzini P , Benuzzi F , Gessaroli G , et al. Epilepsy‐related brain networks in ring chromosome 20 syndrome: an EEG‐fMRI study. Epilepsia. 2014;55(3):403–413. 10.1111/epi.12539 24483620

[50] Hirano Y , Oguni H , Nagata S . Refractory and severe status epilepticus in a patient with ring chromosome 20 syndrome. Brain Dev. 2016;38(8):746–749. 10.1016/j.braindev.2016.02.013 26980640

[51] Ito H , Nagata KI . Functions of CNKSR2 and its association with neurodevelopmental disorders. Cells. 2022;11(2):303. 10.3390/cells11020303 35053419 PMC8774548

[52] Stamberger H , Hammer TB , Gardella E , DRM V , Bertelsen B , Mandelstam S , et al. NEXMIF encephalopathy: an X‐linked disorder with male and female phenotypic patterns. Genet Med. 2021;23(2):363–373. 10.1038/s41436-020-00988-9 33144681

[53] Jeyabalan N , Clement JP . SYNGAP1: mind the gap. Front Cell Neurosci. 2016;10:32. 10.3389/fncel.2016.00032 26912996 PMC4753466

[54] Samanta D . Epilepsy in Angelman syndrome: a scoping review. Brain Dev. 2021;43(1):32–44. 10.1016/j.braindev.2020.08.014 32893075 PMC7688500

[55] Sanchez‐Conde FG , Jimenez‐Vazquez EN , Auerbach DS , Jones DK . The ERG1 K+ channel and its role in neuronal health and disease. Front Mol Neurosci. 2022;15:890368. 10.3389/fnmol.2022.890368 35600076 PMC9113952

[56] Held K , Tóth BI . TRPM3 in brain (Patho)physiology. Front Cell Dev Biol. 2021;9:635659. 10.3389/fcell.2021.635659 33732703 PMC7959729

[57] Mody I , Pearce RA . Diversity of inhibitory neurotransmission through GABAA receptors. Trends Neurosci. 2004;27(9):569–575. 10.1016/j.tins.2004.07.002 15331240

[58] Balestrini S , Galli I , Ricci ML , Parrini E , Mei D , Mastrangelo M , et al. Clinical and genetic landscape of epilepsies with absence seizures and single‐gene etiology. Epilepsia. 2025;67:272–290. 10.1111/epi.18655 41137852 PMC12893263

[59] Zhao X , He Z , Li Y , Yang X , Li B . Atypical absence seizures and gene variants: a gene‐based review of etiology, electro‐clinical features, and associated epilepsy syndrome. Epilepsy Behav. 2024;151:109636. 10.1016/j.yebeh.2024.109636 38232560

[60] Bhatnagar M , Shorvon S . Genetic mutations associated with status epilepticus. Epilepsy Behav. 2015;49:104–110. 10.1016/j.yebeh.2015.04.013 25982265

[61] Onat FY , van Luijtelaar G , Nehlig A , Snead OC . The involvement of limbic structures in typical and atypical absence epilepsy. Epilepsy Res. 2013;103(2–3):111–123. 10.1016/j.eplepsyres.2012.08.008 22989853

[62] Walker MC . Pathophysiology of status epilepticus. Neurosci Lett. 2018;667:84–91. 10.1016/j.neulet.2016.12.044 28011391

[63] Joshi S , Kapur J . Status epilepticus: updates on mechanisms and treatments. Epilepsia Open. 2025;1–15. 10.1002/epi4.70146 PMC13394038 40956087

