# Supplementary material for: Genetic landscape of patients with atypical absence status epilepticus: A systematic review
Source: Epilepsia Open. 2026 May 11;11(4):1111–25. doi: 10.1002/epi4.70275 (PMC13394208; doi:10.1002/epi4.70275)
Supplement: Supplementary file 1 — Data S1 [file EPI4-11-1111-s001.docx]

**Supplementary Material**

**Supplementary Material 1. Research protocol (Systematic review)**

1. **Review Title:** Genetic landscape of atypical absence status epilepticus (AASE): a systematic review.
2. **Authors and contributions:**

| **Name** | **Email** | **Affiliation** | **Principal Investigator** | **Screening** | **Data Extraction** | **Synthesis** |
| --- | --- | --- | --- | --- | --- | --- |
| Maria Cristina Cioclu | mccioclu@gmail.com | 1 |  | x | x | x |
| Giada Giovannini | giovannini.giada@gmail.com | 2 |  | x | x | x |
| Stefano Meletti | stefano.meletti@unimore.it | 1,2 | x |  |  | x |

Affiliations:

1. Department of Biomedical, Metabolic, and Neural Sciences. University of Modena and Reggio Emilia, Modena, Italy
2. Neurophysiology Unit and Epilepsy Centre, Azienda Ospedaliera-Universitaria di Modena, Italy
3. **Support:** No funding was received for this work.
4. **Rationale and Background:** Atypical absence status epilepticus (AASE) is a rare subtype of nonconvulsive status epilepticus (NCSE) without coma, characterized by impaired consciousness and typically continuous or fluctuating slow, generalized epileptiform activity, typically at a frequency < 3 Hz. Only sparse literature exists regarding the genetic diagnoses associated with this electroclinical pattern, mainly across case reports and small case series. Moreover, there is a lack of consensus in the use of terminology and classification across different studies. The main purpose of our study was to review AASE associated with genetic conditions. When available, we also summarized patients’ general clinical features, and reviewed reported treatments and outcomes.
5. **Objectives:**

- **Primary objective:** To identify genetically confirmed diagnoses reported in association with AASE.
- **Secondary objectives:** To describe, when available, the general clinical features, AASE semeiology, EEG features, and reported treatments and status outcomes in genetically confirmed cases.

**METHODS**

1. **Methodology:** we performed a systematic review according to the recommendations of the Preferred Reporting Items for Systematic Reviews and Meta-Analyses (PRISMA)^a^ statement and the Synthesis Without Meta-analysis in systematic reviews (SWiM)^b^ extension.
   1. Page MJ, McKenzie JE, Bossuyt PM, Boutron I, Hoffmann TC, Mulrow CD, et al. The PRISMA 2020 statement: an updated guideline for reporting systematic reviews. BMJ. 2021;372:n71. doi:10.1136/bmj.n71.
   2. Campbell M, McKenzie JE, Sowden A, Katikireddi SV, Brennan SE, Ellis S, et al. Synthesis without meta-analysis (SWiM) in systematic reviews: reporting guideline. BMJ. 2020;368:l6890. doi:10.1136/bmj.l6890.
2. **Eligibility criteria**

| **Study Characteristics** | **Inclusion criteria** | **Exclusion criteria** | **Rationale (if applicable)** |
| --- | --- | --- | --- |
| Population/Participants | Patients of any age, sex, ethnicity with an electroclinical picture consistent with AASE and a confirmed genetic diagnosis. | - Insufficient clinical or EEG data. - Typical absence status epilepticus - Focal features - NCSE in coma - No genetically confirmed diagnosis | NA |
| Age limits of participants | Patients of any age | NA | NA |
| Interventions | NA | NA | NA |
| Exposure of Interest | Patients with a genetic diagnosis; gene variant or chromosomal abnormality defined as pathogenic or likely pathogenic. | - No formal genetic testing. - VUS |  |
| Comparators | NA | NA | NA |
| Study Designs | Case reports, case series, observational studies | Animal and in vitro studies | NA |
| Publication Types  (including grey literature, if applicable) | Full text original articles | narrative reviews, systematic reviews, meta-analyses, editorials, letters, expert opinions, commentaries, surveys, conference abstracts |  |
| Settings | Any healthcare setting | - | - |
| Countries | Patients from any country | - | - |
| Dates | From inception to 15 February 2026 | - | - |
| Languages | English | Non-English articles | - |

1. **Information sources**

The relevant studies were identified through MEDLINE (accessed by PubMed) and EMBASE. The search was last performed on 15 February 2026.

1. **Search strategy**

The search terms were combinations of the following: "atypical absence status epilepticus", "status epilepticus", "non convulsive status epilepticus", “non- convulsive status epilepticus", "NCSE", "non-convulsive” and "gene", "genetics", "epileptic encephalopathy", "developmental and epileptic encephalopathy", "DEE", “genetic epilepsy” in various combinations.

Search strings were:

("absence status epilepticus" OR "atypical absence status epilepticus" OR "absence status") AND ("genetic" OR "genetics" OR "DEE" OR "developmental and epileptic encephalopathy" OR "gene" OR "genes" OR "genetic epilepsy" OR "genetic epilepsies" OR "syndrome" OR "epilepsy syndrome")

("atypical absence" OR "atypical absences" OR "atypical absence seizure" OR "atypical absence seizure") AND ("genetic" OR "genetics" OR "DEE" OR "developmental and epileptic encephalopathy" OR "genetic epilepsy" OR "genetic epilepsies" OR "syndrome" OR "epilepsy syndrome")

("status epilepticus" OR "NCSE" OR "non convulsive" OR "non-convulsive") AND ("genetic" OR "genetics" OR "DEE" OR "developmental and epileptic encephalopathy" OR "epileptic encephalopathy" OR "genetic epilepsy" OR "genetic epilepsies" OR "epilepsy syndrome")

("non convulsive status epilepticus" OR "NCSE" OR "non convulsive" OR "non-convulsive") AND ("genetic" OR "genetics" OR "gene" OR "genetic epilepsy" OR "genetic epilepsies" OR "epilepsy syndrome")

1. **Article selection and screening**

All records retrieved from the electronic databases were imported into Rayyan (Rayyan Systems Inc.). Duplicate records were identified and removed within Rayyan prior to screening. Two reviewers (MCC and GG) independently screened titles and abstracts. Disagreements were resolved by discussion with a third reviewer (SM).

1. **Information and data extraction**

Data were extracted using a predefined standardized form by two authors (MCC, GG) and disagreements were resolved by discussion with a third senior author (SM). We did not contact study investigators for additional information. No automation tools have been used.

The following information was extracted: first author and year of publication, number and demographics of participants, etiologies identified in individual patients, age at AASE onset and latest presentation, semeiology of AASE, EEG, general clinical features especially in terms of epilepsy and neurodevelopmental features, reported treatments and outcomes when available. EEG description data were extracted as reported. Missing information was recorded as not available (NA).

1. **Risk of bias in individual studies and quality assessment**

The risk of bias assessment was not performed, as most included studies were case reports and case series and outcomes were synthesized descriptively.

1. **Synthesis methods**

Given the nature of the included studies, only a descriptive synthesis was performed, and only descriptive statistics were used (frequencies, medians, and ranges). Ages reported in months or years were converted to a common unit for analysis. Results were summarized in descriptive tables. Figures were used to present the PRISMA flow diagram, the distribution of genetic etiologies, and an illustrative testing workflow.

**Supplementary Material 2. Prisma checklist**

| **Section and Topic** | **Item #** | **Checklist item** | **Location where item is reported** |
| --- | --- | --- | --- |
| **TITLE** | | |  |
| Title | 1 | Identify the report as a systematic review. | title |
| **ABSTRACT** | | |  |
| Abstract | 2 | See the PRISMA 2020 for Abstracts checklist. |  |
| **INTRODUCTION** | | |  |
| Rationale | 3 | Describe the rationale for the review in the context of existing knowledge. | Introduction |
| Objectives | 4 | Provide an explicit statement of the objective(s) or question(s) the review addresses. | Lines 100-104, Introduction section |
| **METHODS** | | |  |
| Eligibility criteria | 5 | Specify the inclusion and exclusion criteria for the review and how studies were grouped for the syntheses. | Methods, lines 118-122 and 138-147 |
| Information sources | 6 | Specify all databases, registers, websites, organisations, reference lists and other sources searched or consulted to identify studies. Specify the date when each source was last searched or consulted. | Methods, lines 110-111 |
| Search strategy | 7 | Present the full search strategies for all databases, registers and websites, including any filters and limits used. | Methods, lines 112-117 |
| Selection process | 8 | Specify the methods used to decide whether a study met the inclusion criteria of the review, including how many reviewers screened each record and each report retrieved, whether they worked independently, and if applicable, details of automation tools used in the process. | Methods, lines 148-150 |
| Data collection process | 9 | Specify the methods used to collect data from reports, including how many reviewers collected data from each report, whether they worked independently, any processes for obtaining or confirming data from study investigators, and if applicable, details of automation tools used in the process. | Methods, lines 150-152 |
| Data items | 10a | List and define all outcomes for which data were sought. Specify whether all results that were compatible with each outcome domain in each study were sought (e.g. for all measures, time points, analyses), and if not, the methods used to decide which results to collect. | Methods lines 124-147 |
|  | 10b | List and define all other variables for which data were sought (e.g. participant and intervention characteristics, funding sources). Describe any assumptions made about any missing or unclear information. | Methods lines 153-155 |
| Study risk of bias assessment | 11 | Specify the methods used to assess risk of bias in the included studies, including details of the tool(s) used, how many reviewers assessed each study and whether they worked independently, and if applicable, details of automation tools used in the process. | Not applicable |
| Effect measures | 12 | Specify for each outcome the effect measure(s) (e.g. risk ratio, mean difference) used in the synthesis or presentation of results. | Not applicable |
| Synthesis methods | 13a | Describe the processes used to decide which studies were eligible for each synthesis (e.g. tabulating the study intervention characteristics and comparing against the planned groups for each synthesis (item #5)). | Methods lines 124-147 |
|  | 13b | Describe any methods required to prepare the data for presentation or synthesis, such as handling of missing summary statistics, or data conversions. | Not applicable |
|  | 13c | Describe any methods used to tabulate or visually display results of individual studies and syntheses. | Methods lines 156-158 |
|  | 13d | Describe any methods used to synthesize results and provide a rationale for the choice(s). If meta-analysis was performed, describe the model(s), method(s) to identify the presence and extent of statistical heterogeneity, and software package(s) used. | Methods lines 159-160 |
|  | 13e | Describe any methods used to explore possible causes of heterogeneity among study results (e.g. subgroup analysis, meta-regression). | Not applicable |
|  | 13f | Describe any sensitivity analyses conducted to assess robustness of the synthesized results. | Not applicable |
| Reporting bias assessment | 14 | Describe any methods used to assess risk of bias due to missing results in a synthesis (arising from reporting biases). | Not applicable |
| Certainty assessment | 15 | Describe any methods used to assess certainty (or confidence) in the body of evidence for an outcome. | Not applicable |
| **RESULTS** | | |  |
| Study selection | 16a | Describe the results of the search and selection process, from the number of records identified in the search to the number of studies included in the review, ideally using a flow diagram. | Figure 1 |
|  | 16b | Cite studies that might appear to meet the inclusion criteria, but which were excluded, and explain why they were excluded. | Not applicable |
| Study characteristics | 17 | Cite each included study and present its characteristics. | Results section |
| Risk of bias in studies | 18 | Present assessments of risk of bias for each included study. | Not applicable |
| Results of individual studies | 19 | For all outcomes, present, for each study: (a) summary statistics for each group (where appropriate) and (b) an effect estimate and its precision (e.g. confidence/credible interval), ideally using structured tables or plots. | Results section |
| Results of syntheses | 20a | For each synthesis, briefly summarise the characteristics and risk of bias among contributing studies. | Not applicable |
|  | 20b | Present results of all statistical syntheses conducted. If meta-analysis was done, present for each the summary estimate and its precision (e.g. confidence/credible interval) and measures of statistical heterogeneity. If comparing groups, describe the direction of the effect. | Not applicable |
|  | 20c | Present results of all investigations of possible causes of heterogeneity among study results. | Not applicable |
|  | 20d | Present results of all sensitivity analyses conducted to assess the robustness of the synthesized results. | Not applicable |
| Reporting biases | 21 | Present assessments of risk of bias due to missing results (arising from reporting biases) for each synthesis assessed. | Not applicable |
| Certainty of evidence | 22 | Present assessments of certainty (or confidence) in the body of evidence for each outcome assessed. | Not applicable |
| **DISCUSSION** | | |  |
| Discussion | 23a | Provide a general interpretation of the results in the context of other evidence. | Discussion section |
|  | 23b | Discuss any limitations of the evidence included in the review. | Discussion section, subsection Study Limitations |
|  | 23c | Discuss any limitations of the review processes used. | Discussion section, subsection Study Limitations, lines 399-402 |
|  | 23d | Discuss implications of the results for practice, policy, and future research. | Discussion section, subsection Practical implications and future perspective |
| **OTHER INFORMATION** | | |  |
| Registration and protocol | 24a | Provide registration information for the review, including register name and registration number, or state that the review was not registered. | Methods, line 109 |
|  | 24b | Indicate where the review protocol can be accessed, or state that a protocol was not prepared. | Methods section |
|  | 24c | Describe and explain any amendments to information provided at registration or in the protocol. | Not applicable |
| Support | 25 | Describe sources of financial or non-financial support for the review, and the role of the funders or sponsors in the review. | Acknowledgements  section |
| Competing interests | 26 | Declare any competing interests of review authors. | Disclosures  section |
| Availability of data, code and other materials | 27 | Report which of the following are publicly available and where they can be found: template data collection forms; data extracted from included studies; data used for all analyses; analytic code; any other materials used in the review. | Data availability statement |

*From:*  Page MJ, McKenzie JE, Bossuyt PM, Boutron I, Hoffmann TC, Mulrow CD, et al. The PRISMA 2020 statement: an updated guideline for reporting systematic reviews. BMJ 2021;372:n71. doi: 10.1136/bmj.n71. This work is licensed under CC BY 4.0. To view a copy of this license, visit <https://creativecommons.org/licenses/by/4.0/>

**Supplementary Material 3. Table 1, extended version**

| ***Nr*** | ***Gene/CNV*** | ***Syndrome*** | ***Publication (author, year)*** | ***Nr pts with AASE*** | ***Definition in paper*** | ***Age at first AASE*** | ***Age when SE observed/last SE described*** | ***EEG status*** | ***Clinical semeiology AASE*** | ***Triggers of AASE*** | ***Treatment of AASE*** | ***Outcome of SE*** | ***Seizure type(s)*** | ***Other type(s) of SE*** | ***ID/DD*** | ***Degree of ID/DD*** | ***Drug resistant (yes/no)*** | ***Other feat.*** |  |
| --- | --- | --- | --- | --- | --- | --- | --- | --- | --- | --- | --- | --- | --- | --- | --- | --- | --- | --- | --- |
| 1 | del15q12 | Angelman Syndrome | Matsumoto, 1992 | 4 | AASE | NA, NA, 8y, 6y2mo | NA, NA, 10 yrs, 6y8mo | continuous diffuse 2-3 Hz SW bursts | subseq AA (5-6 sec) with rhythmic E-my | NA | DZP, TRH | 2/4 episodes of AASE resp to DZP, 1/4 resp to TRH, 1/4 NA | AA, My, At, sp, my-At, FS, GTC, FIC, FBTC, hemic. | no | yes | Severe | yes | inappr. laughter, ataxic gait, UL stereotyp |  |
| 2 | del15q11-13 | Angelman Syndrome | Sugimoto, 1992 | 1 | Minor epileptic status | 38 mo | 38 mo | freq irreg. SW | falls, unable to use hands, dulling of consciousness, drooping eyelids, hypersalivation | NA | Clonazepam | marked improv. | GTC, akinetic | no | yes | Severe | NA | severe DD, unable to speak or walk, hyp, strabismus, laughter |  |
| 3 | del15q11-13 | Angelman Syndrome | Laan, 1997 | 3 | ASE | NA | NA | NA | NA | NA | NA | NA | GTC, AA, My, T, FS | GTCSE MySE | NA | NA | NA | NA |  |
| 4 | del15q11-13 | Angelman Syndrome | Minassian, 1998 | 4 | AASE | NA - 36 mo - 15 mo - 12 mo | NA - 36 mo - 15 mo - 12 mo | NA | NA | 1 pt during treatment with PHT and CBZ | NA | NA | AA, T, My, At, GTC | GTCSE, TSE, NCSE | yes | NA | yes | NA |  |
| 5 | UBE3A c.2251insAACTA | Angelman Syndrome | Espay, 2005 | 1 | AASE | 19y | 29 y | bursts (0,2-2,5 sec) rhyth. α, F predom. | change in behav, head ext., EM, up. eye dev., arrest of UL stereotypies | no | NA | resolved after max 2 hours | GTC, AA, At (drop attacks) | no | yes | Severe | yes | ↓HC, full eb, micrognatia, enl. tongue; inappr. laughter, UL stereotyp,tremor, ataxic gait |  |
| 6 | del15q11-13* | Angelman Syndrome | Uemura, 2005 | 10 | AASE | NA | 15 y, 6 y, 6 y, 11 y, 6 y, 7 y, 1 y, 3 y, 2 y, 1 y | NA | NA | NA | NA | NA | FS, My, GTC, Sp, hemic, At | CSE | NA | NA | NA |  |  |
| 7 | del15q11-13* | Angelman Syndrome | Valente, 2006 | 8 | AASE | 5 mo (in 1 pt) | NA | NA | Imp. contact, head dropping or trembling | CBZ in 1 pt, fever in 2 pts | NA | NA | GTC, GT, AA, FIC, Fo, My | MySE | yes | Severe | NA |  |  |
| 8 | UBE3A c.2365del | Angelman syndrome | Melikishvili, 2022 | 2 | NCSE | 66 mo, 49 mo | 66 mo, 49 mo | pt. 1: cont. 1.5–2 Hz S-polyS-SlW; pt. 2: cont. Diff. ↑ amplitude S-polyS-SlW | pt. 1: bad mood, not responsive, poor sleep; pt. 2: ↓ alertness, loss of eye contact, ↓ motor activity | no | IV pyridoxine, BDZ, VPA, KD | resolved with KD | my, FS, At | no | yes | NA | no | DD, happy behav, ataxic gait |  |
| 9 | r(20) | Ring chromosome 20 | Inoue, 1997 | 3 | NCSE/CPSE | 14 y, NA, 7 y | 21 y, 15 y, 31 y | irreg. ↑-volt. SlW, occasional S | clouding of consciousness | Hyperv. for 1 pt | Lidocain in one pt, rest NA | resolved | GTC, AA | NCSE | yes | mild-moderate | yes | NA |  |
| 10 | r(20) | Ring Chromosome 20 | Petit, 1999 | 3 | ASE | 9 y, 5 y, 4 y | NA, NA, 43 y | pt. 1: cont. Diff. Sh theta or SW (>biF); pt 2: fluct. Rhythmic SlW; pt 3 ↑Volt SlW+occasional S | pt 1: loss of contact and motor automatisms; pt 2: confusion, fright, perioral my; pt 3: ↓ motor and verbal spontaneity, peiroral my. | no | NA | spont. Remission | FIC, sleep-rel. szs, GTC | no | yes | NA | yes | behav. disorders |  |
| 11 | r(20) | Ring chromosome 20 | Augustijn, 2001 | 4 | NCSE | 11 y, 14 y, 11y, 8y | 12 y, 14 y, 11.5 y, 8.3 y | gen. epi activity, F-predom | mental slowing | no | NA | NA | TC, FIC | no | yes | NA | yes | subtle nocturnal szs |  |
| 12 | r(20) | Ring chromosome 20 | Shirasaka, 2002 | 1 | AASE | 15 y | 18 y | gen. epi activity, F-predom | drooling, ic | no | DZP | resolved | AA, GTC | no | yes | mild | yes | normal MRI |  |
| 13 | r(20) | Ring chromosome 20 | Locharernkul, 2005 | 2 | CPSE | NA | 25 y, 37 y | prol. gen. rhyth. 3-5 Hz ShW or SlW, few S | fluctuating consciousness, seldom rare my | in some occasions verbal stimuli | PT 1: PHT, VPA; PT 2: VPA | spont. remission (temp. effect of ASM on EEG) | GTC, FIC | no | yes (after epilepsy onset) | NA | yes | NA |  |
|  |  |  |  |  |  |  |  |  |  |  |  |  |  |  |  |  |  |  |  |
| 14 | r(20) | Ring chromosome 20 | Zou, 2006 | 1 | NCSE | NA | 26 y | NA | NA | NA | NA | NA | GTC, Fo, At | no | yes | moderate | yes | hypo, scoliosis, OCD, headache, urinary ret. |  |
| 15 | r(20) | Ring chromosome 20 | Elghezal, 2007 | 1 | NCSE | NA | 12 y | gen ↑-volt. Θ (4-5 Hz), occasional SW | confusional states | NA | NA | NA | T, AA | no | NA | NA | NA | NA |  |
| 16 | r(20) | Ring Chromosome 20 | Alpman, 2007 | 1 | AASE or NCSE | NA | NA | NA | prolonged absence | NA | NA | NA | GTC, FIC, T, AA, At (drop attacks) | NCSE | yes | Moderate-Severe | Yes | behav. disorders, mild dysm |  |
| 17 | r(20) | Ring chromosome 20 | Jacobs, 2008 | 1 | NCSE | 13 y | 13 y | cont. gen 2.5Hz SW, max. F | Prolonged absence | NA | Propofol, PB, MDZ, Thiopental Pentobarbital | super-refractory, lethal | AA, T, Hypermotor, FIC | no | no | / | yes | ADHD, ↓ IQ, left hemip., imp. fine mot. skills |  |
| 18 | r(20) | Ring Chromosome 20 | Vignoli, 2009 | 3 | NCSE | NA | NA, NA, 20 y | cont, gen, bi-F-predom. Sl-SW | cognitive deterioration; aphasia in one patient; ic and slow automatic mov pt nr 3 | NA | VPA+LTG | refract., no recurrence after VPA+LTG | FIC | NA | yes | mild | yes | regr. |  |
| 19 | r(20) | Ring chromosome 20 | Elens, 2012 | 6 | NCSE | NA | 4 y, 8 y, 53 y, 66 y, 22 y, 19 y | NA | behav arrest and ic | NA | NA | NA | T, FIC | no | no | NA | yes | DD, mild dysm. feat., HA |  |
| 20 | r(20) | Ring chromosome 20 | Radhakrishnan, 2012 | 2 | NCSE/CPSE | NA | 15 y, 20 y | gen, rhyth., medium ampl. Θ --> 1.5–3 Hz gen Sl-SW | clouding of consciousness | NA | NA | spont. remission | Fo, C, T, FIC, My, FBTC | no | yes | mild | yes | NA |  |
| 21 | r(20) | Ring chromosome 20 | Vignoli, 2016 | 22 | NCSE | NA | NA | cont. gen SW, F-predom | NA | NA | NA | spont. remission | Fo, FIC, T, EM | no | yes | mild or moderate | 20 DR, 2 seizure free | behav. disorders after sz onset |  |
| 22 | r(20) | Ring chromosome 20 | Bayat, 2022 | 1 | AASE | NA | NA | NA | ic and motor slowdown | NA | NA | NA | AA | NA | yes | profound | yes | Frontal cortical atrophy in MRI |  |
| 23 | r(17) | Ring chromosome 17 | Coppola, 2018 | 1 | NCSE | 28 y | 28 y | subcont. gen SW | no change in behaviour | NA | BDZ | improv. after treatment | GTC, C, T (sleep), FIC | no | yes | moderate | yes | gait imp, non-verbal, imp sphincter control; café-au-lait patches |  |
| 24 | r(17) | Ring chromosome 17 | Ricard-Mousnier, 2007 | 1 | diurnal ESE | 3 y | 4 y | ge Sl-SW central-predom (0,5-3 Hz); hours | ic and motor slowdown | NA | NA | spont. remission | GTC, T, C, At | no | yes | moderate | yes | ↓HC, GR |  |
| 25 | 4p- | 4-p syndrome | Battaglia, 2003 | 1 | AASE | 2y6mo | NA | NA | ic and motor slowdown | NA | NA | NA | GTC, FS (C), MyA | no | yes | Severe | yes | NA |  |
| 26 | 4p- | 4-p syndrome | Valente, 2003 | 1 | AASE | 30 mo | 6 y | NA | ic and motor slowdown | NA | NA | not refractory | unilateral My, AA, GTC | MySE | yes | Severe | no | dysm. feat, feeding difficulties, GR, hypo |  |
| 27 | CNKSR2 | CNKSR2-DEE | Bonardi, 2020 | 2 | ASE | 7y6mo,8 y | 11y, 8y | NA | ic, fluctuating E-My and distal limbs | NA | NA | NA prolonged | My, AA, GTC | ESES | yes | Moderate-Severe | one yes and one no | regr., stereotyp, speech and facial dyspraxia, HA |  |
| 28 | KCNH2 | KCNH2-DEE | Ghimire, 2022 | 1 | Absence epilepsy with Status Epilepticus | 11 y | 11 y | occasional, gen S and poly-SW | NA | NA | ESM and ZNS | resolved | AA | no | yes | mild | no | ASD, ADHD |  |
| 29 | NEXMIF | NEXMIF-DEE | Ogasawara, 2020 | 1 | NCSE | 38 y | 38 y | gen S, poliS, ShW | ic, staring, occasional E-My | NA | NA | NA | GTC | no | yes | mild | no | mild dysm. facial feat., obesity |  |
| 30 | NEXMIF | NEXMIF-DEE | Wu, 2020 | 1 | AASE | 26 y | 29 y | 1.5–2.5 Hz semi-rhyth. gen. SW; eye-closure sensitivity | ↓ responsiveness, mydriasis, E-My | NA | MDZ | Resolved with MDZ | AA, GTC | no | yes | mild | yes | minor dysm. feat., diabetes |  |
| 31 | NEXMIF | NEXMIF-DEE | Cioclu, 2021 | 1 | NCSE | 9 y | 28 y | cont., gen. S, poly-SW, eye closure-sensitivity; worse with HV and IPS | ic | NA | LZP* | Resolved with LZP, recurrent | NCSE, GTC | no | yes | mild | yes | borderline IQ |  |
| 32 | TRPM3 | TRPM3-DEE | Kang, 2021 | 1 | AASE | 7y2mo | 7y2mo | NA | NA | NA | NA | NA | Sp, T | no | yes | Severe | yes | mild hypo, dysm, feat. |  |
| 33 | SYNGAP1 | SYNGAP1-DEE | Lo Barco, 2021 | 1 | AASE | NA | NA | eye-closure sensitivity and FOS | ic | NA | NA | NA | My, At, AAAt, T | NA | yes | Severe | no | ASD, ↓ pain sensit., hyperacusis, ↓HC |  |
| 34 | GABRB1 | DEE45 | Monfrini, 2023 | 1 | AASE | NA | 14 y | prolonged 2 Hz SW | NA | Respiratory infection | NA | NA | Fo, Fo migr, sp, T | no | yes | profound | yes | Acquired ↓HC, tetraparesis, dysphagia, restrictive pulmonary syndrome, OSAS |  |
